# Supplementary material for: Novel CDK12/13 Inhibitors AU-15506 and AU-16770 Are Potent Anti-Cancer Agents in EGFR Mutant Lung Adenocarcinoma with and without Osimertinib Resistance
Source: Cancers (Basel). 2023 Apr 12;15(8):2263. doi: 10.3390/cancers15082263 (PMC10137182; doi:10.3390/cancers15082263)

Supplementary Figure S1: Copy number assay for *ERBB2 (HER2) and MET* genes

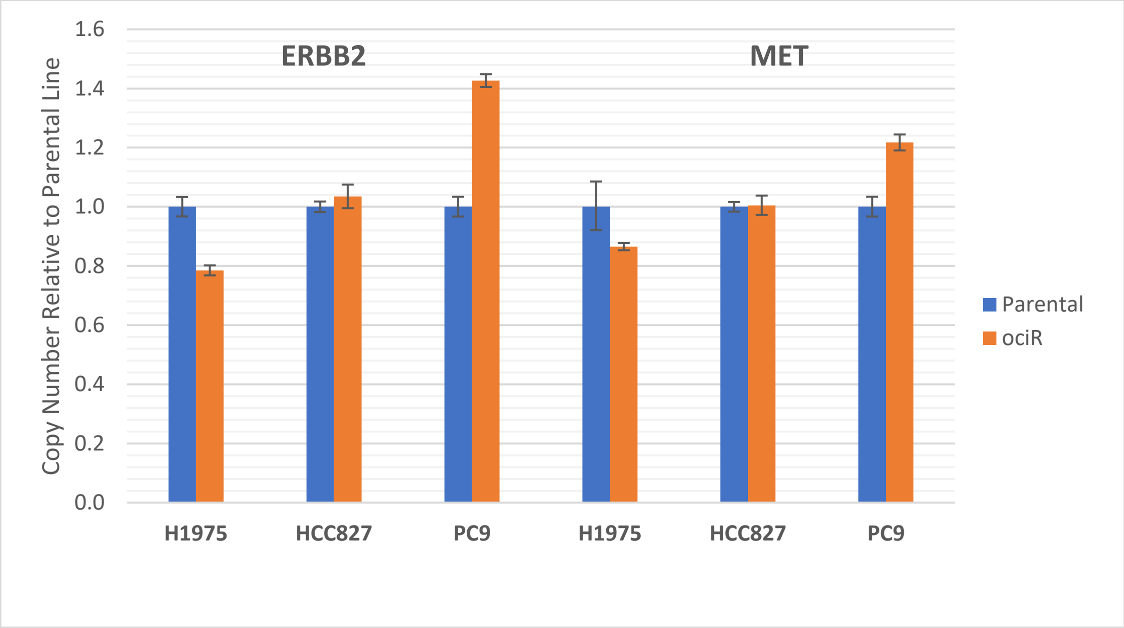

Supplementary Figure S2: Selectivity profile of Aurigene lead compounds AU-CBB-16770 and AU-CBB-15506 in short kinase panel assay

Kinase Panel	AU-16770		AU-15506	
	% Inhibition (µM)			
	1	10	1	10
ALK	0	-11	8	12
c-SRC	-1	-4	5	1
Flt3	-4	34	20	35
Flt4	0	-4	3	2
JAK2	-4	2	8	-5
KDR(VEGR2)	4	38	16	33
ZAP70	-12	4	1	1
TrkA	-1	38	12	32
Aur A	11	33	18	38
INSR	5	8	-3	-11
PDGFR-β	1	0	1	6
p38-α	1	9	1	3
AXL	1	1	0	-19
IRAK4	7	8	-2	-1
GLK	26	31	14	16
MUSK	6	6	21	-1
EGFR	-4	-4	-4	7
FAK	2	11	5	4
PI3K-γ	0	7	-3	1
cMET	-3	-5	-5	-8
FGFR1	42	77	12	47
RON	-1	8	21	2
ACK1	4	7	-8	-2
PKC-α	4	35	1	23
Tie2	-5	-1	3	3
SGK1	17	41	23	40

Supplementary Figure S3

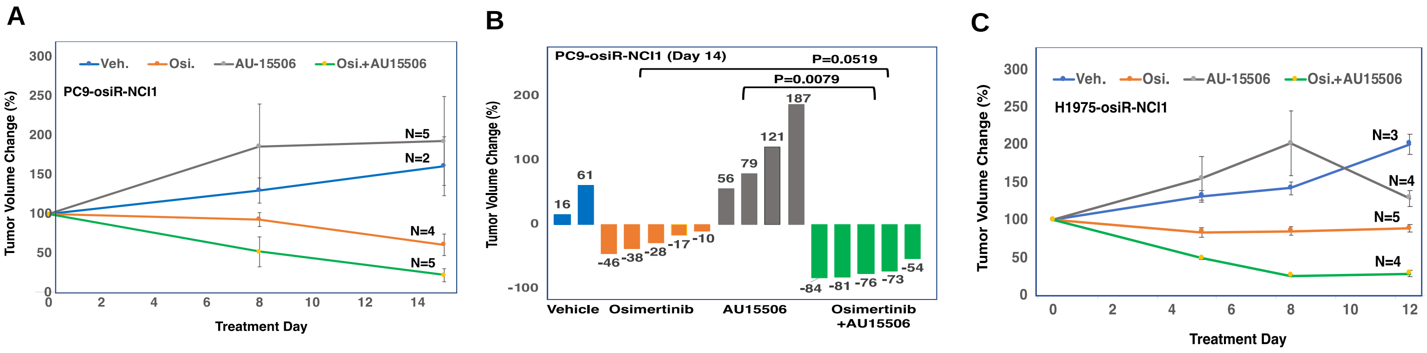

AU15506 in combination with Osimertinib inhibits xenograft tumor growth. Mice bearing PC9-osiR-NCI1-derived tumors were treated for 14 days with vehicle (N = 2), 5 mg/kg/day osimertinib (N = 4), 5 mg/kg/day AU-15506 (N = 5), or AU-15506-osimertinib combination (N = 5). A. Tumor volume was measured daily and percent change per treatment group plotted. B. Bar plot of percent tumor volume change for each mouse on day 14 shows that overall tumor burden is significantly reduced in mice receiving AU-15506-osimertinib combination treatment. C. Mice bearing H1975-osiR-NCI1-derived tumors were treated for 12 days with vehicle (N = 3), 5 mg/kg/day osimertinib (N = 5), 5 mg/kg/day AU-15506 (N = 4), or AU-15506-osimertinib (N = 4). Plot of daily percent tumor volume change shows AU-15506-osimertinib combination treatment reduces tumor size.

Supplementary Figure S4: Relative abundance (%) of CHK1 and CHK2 proteins in osimertinib resistant PC9-osiR-NCI1 cells treated with DMSO and AU16770

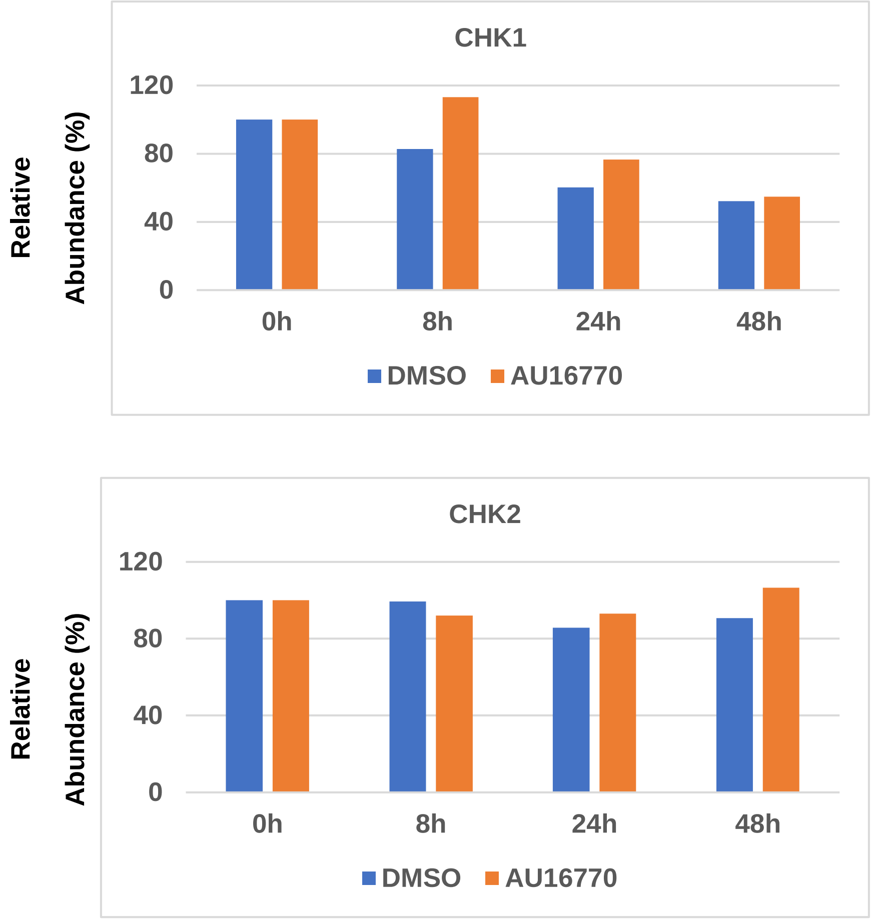

Supplement: Supplementary file 1 [file cancers-15-02263-s001.zip › cancers-2339073-supplementary.pdf]
